# The Metropolitan Hospital

**Published:** 1895-06-08

**Authors:** 


### The Metropolitan Hospital.

Mr. Leopold de Rothschild has circulated the following appeal on behalf of this hospital. The generosity and invaluable kindness of the Rothschild family, and especially of Mr. Leopold de Rothschild, should ensure a most generous response to this appeal. Few can fail to desire to send a cheque in such circumstances, and we hope this sum sent to Mr. de Rothschild will make this dinner one of the most memorable of the year 1895. The letter is as follows: "As I shall have the honour of presiding at the festival dinner of the Metropolitan Hospital on Thursday, June 13th, at the Whitehall Rooms, I venture to hope that you will peruse the enclosed report, which will briefly give you some particulars of the excellent work done by this institution; and I will only add that during last year 781 in-patients were under treatment, and the out-patients' attendances reached the enormous total of 78,238. I rely on your generosity to respond to my appeal on behalf of a hospital which has claims upon the City, the charitable public, and especially the Jewish community.—Yours very truly, Leopold de Rothschild."

